# Identification of microRNA-Like RNAs in the Filamentous Fungus *Trichoderma reesei* by Solexa Sequencing

**DOI:** 10.1371/journal.pone.0076288

**Published:** 2013-10-02

**Authors:** Kang Kang, Jiasheng Zhong, Liang Jiang, Gang Liu, Christine Yuan Gou, Qiong Wu, You Wang, Jun Luo, Deming Gou

**Affiliations:** 1 College of Life Sciences, Shenzhen Key Laboratory of Microbial Genetic Engineering, Shenzhen University, Shenzhen, China; 2 Department of Marine Biology, Shenzhen Key Laboratory of Marine Biotechnology and Ecology, Shenzhen University, Shenzhen, China; 3 College of Animal Science and Technology, Northwest A&F University, Yangling, Shaanxi, China; 4 Northwestern University, Evanston, Illinois, United States of America; New England Biolabs, Inc., United States of America

## Abstract

microRNAs (miRNAs) are non-coding small RNAs (sRNAs) capable of negatively regulating gene expression. Recently, microRNA-like small RNAs (milRNAs) were discovered in several filamentous fungi but not yet in *Trichoderma reesei*, an industrial filamentous fungus that can secrete abundant hydrolases. To explore the presence of milRNA in *T. reesei* and evaluate their expression under induction of cellulose, two *T. reesei* sRNA libraries of cellulose induction (IN) and non-induction (CON) were generated and sequenced using Solexa sequencing technology. A total of 726 and 631 sRNAs were obtained from the IN and CON samples, respectively. Global expression analysis showed an extensively differential expression of sRNAs in *T. reesei* under the two conditions. Thirteen predicted milRNAs were identified in *T. reesei* based on the short hairpin structure analysis. The milRNA profiles obtained in deep sequencing were further validated by RT-qPCR assay. Computational analysis predicted a number of potential targets relating to many processes including regulation of enzyme expression. The presence and differential expression of *T. reesei* milRNAs imply that milRNA might play a role in *T. reesei* growth and cellulase induction. This work lays foundation for further functional study of fungal milRNAs and their industrial application.

## Introduction

microRNAs (miRNAs) are small non-coding RNAs of 18~25 nucleotides that negatively regulate gene expression by binding to the target mRNAs [1]. Mature miRNAs are processed from primary miRNA transcripts (pri-miRNA) by the endonuclease Drosha, producing a precursor hairpin structure of 60~70 nucleotides, termed pre-miRNAs. The pre-miRNAs are exported from nucleus to cytoplasm, where they are further cleaved by an endonuclease, Dicer, to yield mature miRNAs [2,3]. As a component of RNA-induced silencing complex (RISC), mature miRNAs guide the binding of RISC to mRNA targets, forcing mRNA degradation and/or translational inhibition [4].

Since the discovery of the first miRNA *lin-4* in *Caenorhabditis elegans* [5], miRNAs have been identified in diverse organisms including animals, plants, and unicellular eukaryotes such as algae [6], *Giardia lamblia* [7], and *Trichomonas vaginalis* [8]. The presence of miRNAs in the unicellular organisms suggested that the miRNA pathway is an ancient mechanism of gene regulation [9]. Filamentous fungi are an important group of multicellular eukaryotes with over one billion years of evolution [10]. A variety of small RNAs (sRNAs) and its mediated RNA interference (RNAi) have been shown in ﬁlamentous fungi [11,12]. Recently, several groups reported that miRNA-like sRNAs (milRNAs) also exist in the filamentous fungi, including *Neurospora crassa* [13], *Sclerotinia sclerotiorum* [14] and *Metarhizium anisopliae* [15]. The success of siRNA-mediated gene silencing in *T. reesei* [16,17], an important industrial fungus capable of secreting a large amount of cellulolytic enzymes, suggests the RISC machinery and implies the presence of milRNAs in this fungus. However, neither miRNAs nor milRNAs have been reported in *T. reesei*, raising the question as to whether miRNAs exist in *T. reesei*.

Our purpose in this work is to explore the existence of milRNA in *T. reesei* and characterize their expression profile under cellulase induction. Two sRNA libraries of cellulose induction (IN) and non-induction (CON) were generated and sequenced using high-throughput sequencing technology.

## Materials and Methods

### *T. reesei* sample preparation

*T. reesei* strain QM9414 (ATCC 26921) were grown on potato dextrose agar (PDA) to obtain conidia. A total of 1×10^7^conidia were inoculated into 25 ml of basal medium supplemented with 2% glucose at 28°C for 24 h with shaking at 250 rpm. The basal medium contains 0.4% KH_2_PO_4_, 0.28% (NH4)_2_SO_4_, 0.06% MgSO_4_, 0.08% CaCl_2_·2 H_2_O, 0.0005% FeSO_4_·7 H_2_O, 0.00016% MnSO_4_·H_2_O, 0.00017% ZnSO_4_·7 H_2_O, 0.00037% CoCl_2_·6H_2_O, 0.2% peptone and 0.1% Tween-80. For induction cultivation (IN), 2.5 ml mycelium supernatant was added into 50 ml of basal medium supplemented with 3% Avicel (Sigma PH101) at 28°C for 96 h. The same amount of mycelium supernatant was added into 50 ml of basal medium supplemented with 2% glucose for 72 h, which served as a non-induction control (CON).

### Cellulase activity assays

The cellulase filter paper activity of *T. reesei* culture supernatant was measured with a method provided by Ghose [18].

### RNA extraction and Solexa sequencing

The total RNAs of *T. reesei* IN and CON mycelium were extracted using the mirVana PARIS Kit (Ambion) according to the manufacturer’s instruction. sRNAs of *T. reesei* between 18~30 nucleotides were isolated using denatured polyacrylamide gel electrophoresis (PAGE). After ligated to 5’ and 3’ adapters, the sRNAs were reverse transcribed to cDNA using RT-PCR reaction. The PCR products were sequenced with an Illumina Genome Analyzer (BGI, Shenzhen, China).

### Sequencing data analysis

The final clean reads were obtained by getting rid of the contaminant reads with 5' primer contaminants or polyA and those without 3' primer or insert tag. The sRNA sequences were mapped to the *T. reesei* genome [19] (http://genome.jgi-psf.org/Trire2/Trire2.home.html) using the Short Oligo Alignment Program (SOAP). We used the Rfam database (10.1) to eliminate the sRNAs originated from rRNA, tRNA, snRNA and snoRNA. The hairpin structures of the sRNAs were analyzed using the online tool Mfold [20] (http://mfold.rna.albany.edu/?q=mfold/RNA-Folding-Form). Briefly, the sRNA sequence, as well as the 100 upstream nucleotides and 100 downstream nucleotides were folded using the Mfold software. The minimal free energy (MFE) of the hairpin structure was set as -15 kcal mol^-1^.

### Globally differential expression of sRNAs

The global comparison of sRNA expression between the IN and CON samples was carried out. The procedure is as follows: (1) Normalize the expression of sRNAs in IN and CON samples to get the expression of transcript per million (TPM). Normalization formula: Normalized expression = actual miRNA count/total count of clean reads*1,000,000; (2) Calculate the log_2_ fold change (IN/CON); (3) evaluate the *P*-value using Fisher exact test; (4) graph a volcano plot that shows statistical significance versus fold change of expression on the y- and x-axes, respectively. *P*-values <0.01 were considered statistically significant. sRNAs were divided into three groups: (1) up-expressed sRNAs: log_2_ fold change≥1.0; (2) equally expressed sRNAs: -1.0<log_2_ fold change<1.0; (3) down-expressed sRNAs: log_2_ fold change ≤ -1.0. False discovery rates (FDR) were estimated, which was carried out by R statistical package version 3.0.1 (downloaded from http://www.r-project.org/). milRNAs with FDR< 0.05 for their expression in regression models were defined as differentially expressed miRNAs.

### Analysis of milRNA expression using RT-qPCR method

We evaluated the expression of *T. reesei* milRNAs using the S-Poly(T) RT-qPCR method as described [21]. The 18S rRNA was used as a normalization control and the CON was served as the reference sample. The comparative Ct method (ΔΔCt) was exploited to calculate the relative expression levels of milRNAs. The probe and primers used were listed in Table S1. All analyses were performed in biological triplicate. Statistical analysis was performed with GraphPad Prism 5 using a two-tailed Student’s t-test. A *P*-value<0.05 was considered statistically significant.

### milRNA target prediction and data analysis

The miRanda program was used to predict the potential targets of the *T. reesei* milRNAs. The parameters were set as follows: a gap opening penalty of 8; a gap extension penalty of 2; a score threshold of 90; an energy threshold of -23 kcal mol^-1^; and a scaling parameter of 2 [15]. We used the 1,000 bp sequences downstream of stop codon of all genes in *T. reesei* genome as database for the prediction analysis (http://genome.jgi-psf.org/Trire2/Trire2.home.html) due to the lacking of the 3’ UTR sequence database in *T. reesei*. The Gene Ontology (GO) annotations for predicted protein targets were available on JGI by search with protein ID (TreeseiV2_FrozenGeneCatalog20081022), and followed by functional classification using the WEGO software [22] (http://wego.genomics.org.cn/cgi-bin/wego/index.pl).

### GEO accession numbers

The data obtained from sRNA deep sequencing studies were deposited in the Gene Expression Omnibus database at NCBI (http://www.ncbi.nlm.nih.gov/geo/). The accession number for GEO is GSE46679.

## Results and Discussion

### Overview of *T. reesei* sRNAs in Solexa sequencing

Accumulating evidence indicates the critical regulatory roles of miRNA in various biological processes in animals and plants. Recently, microRNA-like sRNAs (milRNAs) were discovered in fungi *N. crassa* [13], *S. sclerotiorum* [14] and *M. anisopliae* [15]. *T. reesei* is another important industrial fungus while miRNAs or milRNAs have not yet been discovered. To explore the existence of milRNAs in *T. reesei*, two separate *T. reesei* sRNA libraries were generated. First, *T. reesei* was cultivated in basal medium supplemented with 3% Avicel (IN) or 2% glucose (CON), respectively. The cellulase activities of the culture supernatant were measured and the results showed that the IN sample had a significant filter paper activity of approximately 1.10 FPU/ml, while the CON sample had no detectable activity (Figure S1). Next, the mycelium of both samples was subjected to RNA extraction. The quality of the RNA was measured with the Agilent 2100 Bioanalyzer (Table S2). Finally, two sRNA libraries of 18~30 nucleotides from IN and CON samples were prepared and sequenced using Solexa high-throughput sequencing technology.

**Figure 1:**
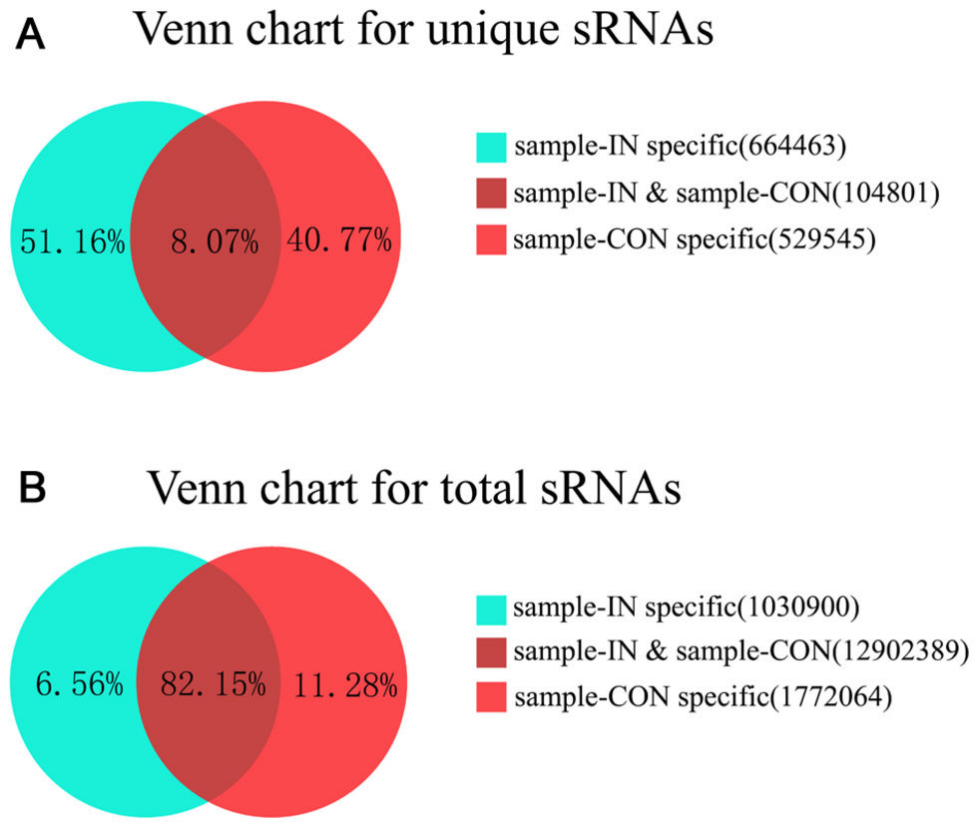
Common and specific sequences between *T. reesei* IN and CON samples. Venn charts show the summary of unique (A) and total (B) sRNAs sequence between *T. reesei* IN and CON samples. The number in brackets indicates the reads of the sequences.

After removing the low quality and adaptor sequences, 7,519,676 and 8,185,677 clean reads were obtained, which represented 664,463 and 529,545 unique sRNA sequences for *T. reesei* IN and CON sample, respectively (Table 1, Figure 1). The comparative analysis showed that the common “unique sRNAs” between the two samples were 8.07% (104,801), which constituted 82.15% (12,902,389) of the total sRNAs (Figure 1). The large number of sRNA sequences corresponding to a minority of the reads in only IN or CON samples suggested that *T. reesei* has a high degree of sRNA sequence complexity.

**Table 1:** Statistical summary of sRNAs from *T. reesei* IN and CON samples.

	**Reads**	**%**
**IN**		
Total reads	8,040,063	
High quality	7,970,582	100%
3' adapter null	6,855	0.09%
Insert null	4,447	0.06%
5' adapter contaminants	22,916	0.29%
Smaller than 18 nt	416,557	5.23%
PolyA	131	0.00%
Clean reads	7,519,676	94.34%
**CON**		
Total reads	8,640,163	
High quality	8,595,187	100%
3' adapter null	5,601	0.07%
Insert null	4,239	0.05%
5' adapter contaminants	7,301	0.08%
Smaller than 18 nt	392,333	4.56%
PolyA	36	0.00%
Clean reads	8,185,677	95.24%

All of the unique sequences were then compared with *T. reesei* genome, and the sequences that perfectly matched to the genomes were used for further analysis. The majority of sRNAs in IN and CON samples was 19~24 nucleotides in length (Figure S2). The length of sRNA peaked at 21 nucleotides in the IN library opposed to 19 nucleotides in the CON library, indicating a different predominance of sRNAs in length between the two samples. By aligning the sequences to the Rfam database, a total of 62,755 and 53,881 unique sRNAs from IN and CON samples, which originated from 50,338 and 46,267 rRNA, 10,072 and 6,157 tRNA, 1,981 and 1,340 snRNA, and 364 and 117 snoRNA, respectively, were removed (Figure S3). By doing a sequence alignment among the remaining sRNAs, many species of sRNAs were recognized as the truncated copies of the same sRNA molecule, with the sequence in the highest count representing the sequence of this sRNA. Finally, 726 and 631 sRNAs were obtained from IN and CON samples, respectively, which were subjected to analysis of global expression profile and RNA secondary structure.

### Global expression analysis of *T. reesei* sRNAs

**Figure 2:**
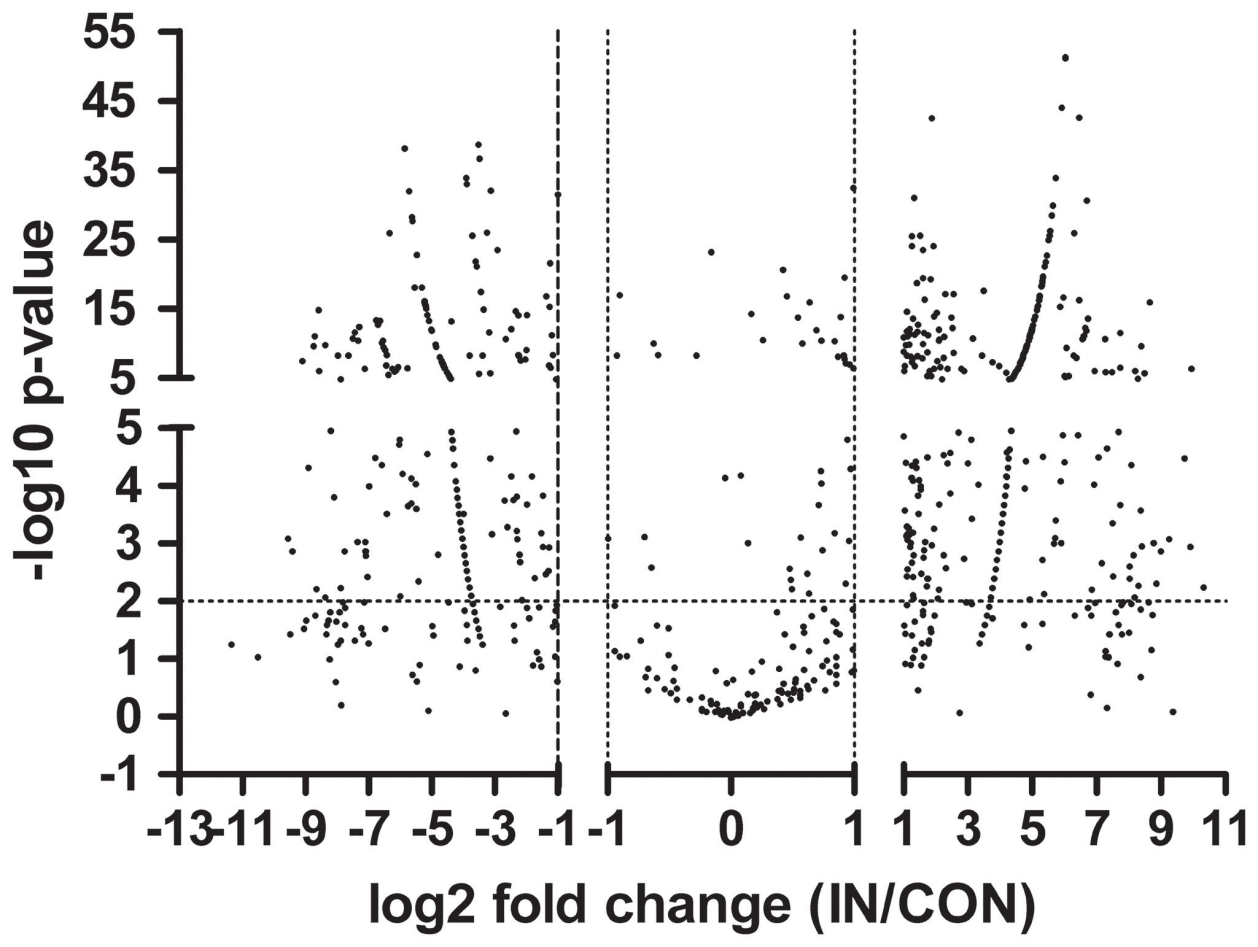
Global expression analysis of *T. reesei* sRNAs. A volcano plot shows the magnitude (fold change; x-axis) and significance (*P*-value; y-axis) of the unique sRNAs between IN and CON samples. The horizontal dashed line indicates the threshold of statistical significance (*P*-value= 0.01). The vertical dashed line shows the fold changes of 2.0 (log_2_ = ±1.0). sRNAs were divided into three groups based on the fold changes (IN/CON) : (1) up-expressed sRNAs: log_2_ fold change≥1.0; (2) equally expressed sRNAs: -1.0<log_2_ fold change<1.0; (3) down-expressed sRNAs: log_2_ fold change ≤ -1.0.

The differential expression of sRNAs between IN and CON samples were analyzed. The fold change of normalized expression of IN sample to normalized expression of CON sample was calculated for each sRNA. We set the fold change of 2.0 (log_2_ = ±1.0) as a cut-off and divided the sRNAs into three groups: (1) up-expressed sRNAs: log_2_ fold change≥1.0; (2) equally expressed sRNAs: -1.0<log_2_ fold change<1.0; (3) down-expressed sRNAs: log_2_ fold change ≤ -1.0. Among the sRNAs analyzed, there were 513 up-expressed sRNAs, 167 equally expressed sRNAs and 312 down-expressed sRNAs. The dramatic sRNA expression changes under different growth conditions indicate that sRNAs might play important roles in cellulose induced cellulase production (Figure 2).

### Identification of milRNAs in *T. reesei*

sRNAs were recognized as potential miRNAs when they were able to form hairpin structure with flanking nucleotide sequences in the genome [23]. To identify the milRNA in *T. reesei*, we used an online tool Mfold to analyze the hairpin structure of the sRNAs in both samples. Setting the criteria is of great importance in miRNA secondary structure prediction. Many criteria have ever been used for the miRNA prediction including the base pairs in a stem, the lengths of the whole hairpin and the hairpin loop, the minimal free energy (ΔG), etc [24]. However, the structures of pre-milRNAs in filamentous fungi might be different from those in animals and plants. For example, the length of pre-milRNAs was about 38 ~ 160 nucleotides in *N. crassa* which possesses a bigger range than that in plants [13]. In this work, we mainly focused on the length of the stem and the ΔG criteria during milRNA prediction. Additionally, GU is a wobble base pair with comparable thermodynamic stability to a Watson-Crick base pair. Thus, the GU base pair was permitted in the Mfold criteria as performed [25].

**Figure 3:**
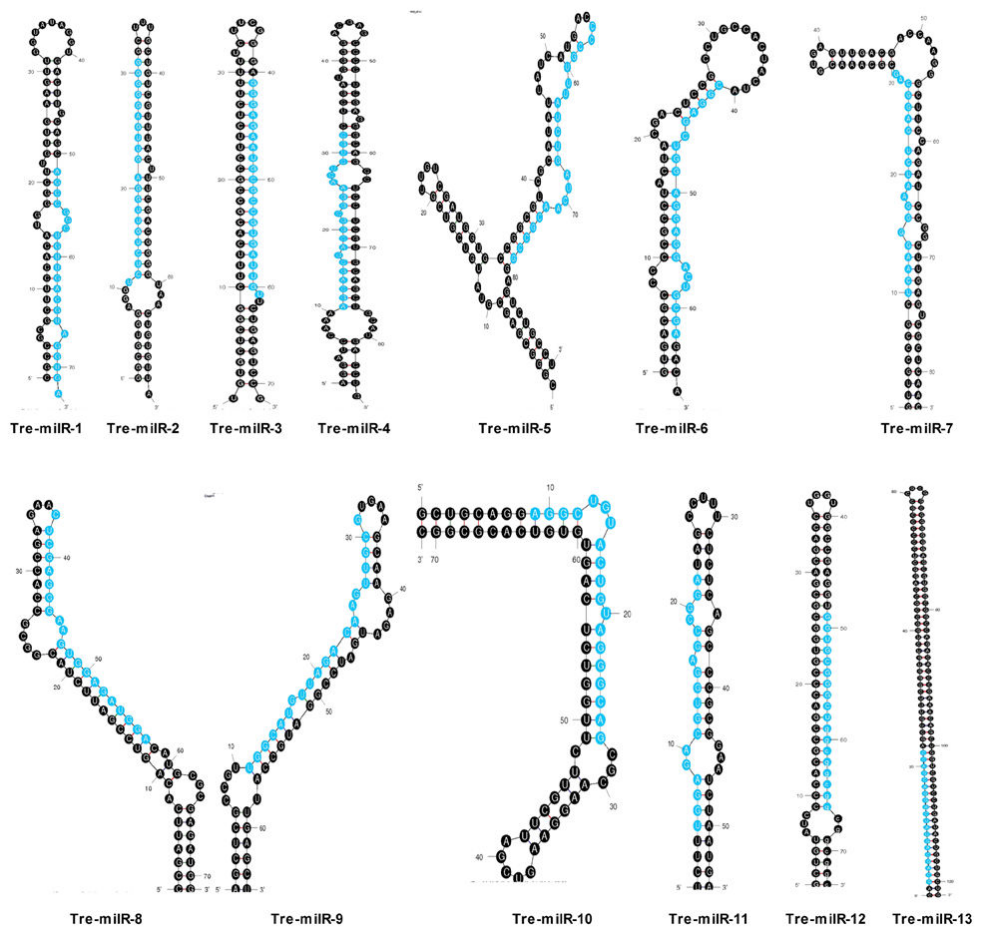
Prediction of *T. reesei* pre-milRNAs hairpin structures. The secondary structures were analyzed with the Mfold software. The mature milRNA sequences were highlighted in blue.

Thirteen candidate milRNAs were identified in *T. reesei* (Figure 3). The lengths of these *T. reesei* pre-milRNAs were similar to those in plants and animals [24] but much shorter than those reported in *N. crassa* [13]. The number of predicted milRNAs in *T. reesei* is much less than that in animals and plants, but is close to that in filamentous fungi. For example, four milRNAs were presented in *N. crassa* [13]. Two milRNAs and forty-two candidate milRNAs were reported in *S. sclerotiorum* [14], and fifteen in *M. anisopliae* [15]. Furthermore, no miRNA-miRNA* duplex was observed in *T. reesei*, which was yet found in a plant pathogenic fungus *S. sclerotiorum* [14]. Sequence search of *T. reesei* milRNAs on miRBase found no homologues in any other organisms including filamentous fungi, indicating a species-specific feature of *T. reesei* milRNAs.

The biogenesis of miRNAs is a multistep process, undergoing the pri-miRNA, pre-miRNA, and mature miRNA steps. Lee demonstrated that *N. crassa* generated milRNA by at least four different pathways involving components of Dicers, QDE-2, the exonuclease QIP, and an RNase III domain-containing protein, MRPL3 [13]. A homology search of the components in miRNA biogenesis showed that there were two Dicer-like and three Argonaute-like proteins in *T. reesei* genome (Table S3), providing evidences for the milRNA existence in this fungus. We thus termed the milRNAs in *T. reesei* as Tre-milRNAs concordant with the nomenclature used in *N. crassa* [13].

### Differential expression of *T. reesei* milRNAs under different growth conditions

Another purpose of this study is to explore the role of milRNAs involved in the *T. reesei* cellulase production. We analyzed the Tre-milRNA expressions between IN and CON samples. Among the thirteen milRNAs, six were IN-specific, one was unique in CON, and six were present in both samples (Table 2). Of the six common milRNAs, Tre-milR-3, Tre-milR-5, Tre-milR-10 and Tre-milR-13 were up-regulated while Tre-milR-7 and Tre-milR-8 were down-regulated in IN sample when compared with that in CON sample. Among them, Tre-milR-5 and Tre-milR-10 were in high abundance in both samples, whereas other milRNAs expressed at a relatively low level. The different expression patterns of milRNAs suggested that milRNAs might be associated with the *T. reesei* metabolic processes, specifically with the cellulase induction.

**Table 2:** *T. reesei* milRNAs identified by Solexa sequencing.

milRNA	Sequence (5’–3’)	Length (nt)	Total reads		Location of milRNAs			MFE (kcal mol^-1^)	*P*-value	FDR
			IN	CON	Scaffold	Start	End			
Tre-milR-1	AGCCGGCTGTTGACGTAGGTGA	22	5	0	16	434562	434583	-22.6	7.36E-03	2.94E-02
Tre-milR-2	TCTCTGTTGGAGTTGAGGGGG	21	9	0	4	95498	95478	-23.3	1.27E-03	1.52E-03
Tre-milR-3	GGGAGAATGCGCCGTGATTGT	21	100	53	20	508320	508340	-31.3	3.47E-03	3.93E-03
Tre-milR-4	AGCAGCGACGGCGGAACTCTGC	22	1,018	0	8	423216	423195	-38.0	2.69E-27	1.33E-15
Tre-milR-5	CCCGTTTATCTGATCAACGCCG	22	4,486	1,306	26	411357	411336	-23.5	7.50E-07	1.55E-06
Tre-milR-6	CGGAGCTGGAGGAGGACTGCGA	22	56	0	22	244532	244511	-17.5	2.95E-20	1.40E-15
Tre-milR-7	TCAAGGGGAATCTGAGGCAG	20	112	946	1	494569	494550	-29.5	1.22E-06	2.10E-06
Tre-milR-8	CTCGAGGGAAGTGGAGATGGA	21	22	55	6	551579	551599	-23.5	6.21E-04	7.71E-04
Tre-milR-9	TGGCATGTTAGACAAGTTGCG	21	6	0	7	628677	628695	-18.6	4.22E-03	1.69E-02
Tre-milR-10	AGGCTGTACTGTAGGGCAG	19	1,386	839	1	984928	984948	-20.2	1.26E-03	1.49E-03
Tre-milR-11	TGGAGACGTGGAGCCGGA	18	0	23	9	154864	154881	-15.5	3.23E-07	5.86E-07
Tre-milR-12	GGTGCGGGCTGGCGGCGG	18	54	0	13	231019	231036	-36.2	4.67E-17	1.47E-15
Tre-milR-13	CCAGCAGGACTATGACGACG	20	23	6	18	372972	372953	-138.8	6.35E-04	8.13E-04

MFE: minimal free energy; FDR: false discovery rates.

**Figure 4:**
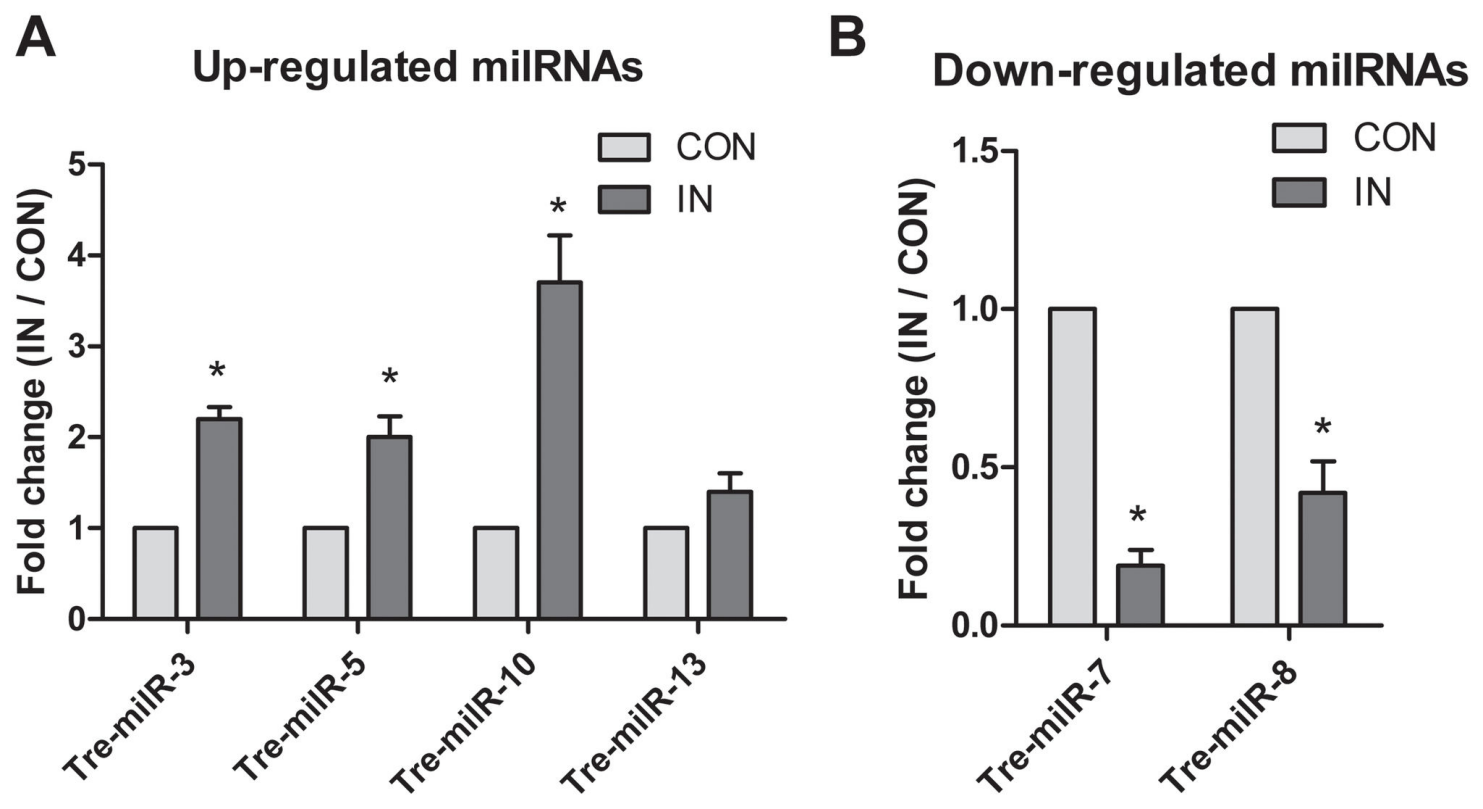
Validation of differentially expressed milRNAs obtained in high-throughput sequencing with S-Poly(T) RT-qPCR method. The expression levels of the selected milRNAs were assayed in both IN and CON. The 18S rRNA was used as a normalization control and the CON was served as the reference sample. All analyses were performed in biological triplicate. Statistical analysis was performed using a two-tailed Student’s t-test. Each bar represents mean ± SD. *P<0.05.

We further validated the level of milRNAs that were present in both samples with RT-qPCR assay. As shown in Figure 4A, the expression of Tre-milR-3, Tre-milR-5, Tre-milR-10 and Tre-milR-13 in IN sample resulted in a 2.2-, 2.0-, 3.7- and 1.4-fold up-regulation, respectively, when compared with that in CON sample. Moreover, Tre-milR-7 and Tre-milR-8 were 5.2- and 2.4-fold down-regulated in IN relative to CON, respectively (Figure 4B). The results of the RT-qPCR assay showed a good coincidence with the data obtained by high-throughput sequencing.

### Target gene prediction of *T. reesei* milRNAs

We applied the miRanda software to predict the potential targets of *T. reesei* milRNAs. Since there was no *T. reesei* 3’ UTR database available, we chose the 1,000 bp sequences downstream of the stop codon of all genes in *T. reesei* genome as a hypothetical 3’ UTR database. The result showed that *T. reesei* milRNAs except Tre-milR-10 could bind to at least one target (Table S4). Surprisingly, Tre-milR-6 was able to match 101 target genes. Intriguingly, Tre-milR-4, an IN-specific milRNA, was predicted to target Cre1, a carbon catabolite repressor. The effects of Cre1 upon the negative regulation of cellulases and hemicellulases expression in *T. reesei* had been extensively reported [26-29]. Whether the significantly expression of Tre-milR-4 under inductive condition promotes the production of cellulases via targeting Cre1 needs further experimental study.

The WEGO software was used to perform a Gene Ontology (GO) analysis for functional classification of the predicted targets [22]. The result showed that the predicted targets were involved in different biological processes including substrate binding and catalysis, enzymatic transcriptional and translational regulation, transportation, growth, pigmentation, localization, response to stimulus and many other metabolic process (Figure S4).

## Conclusion

In present work, thirteen predicted milRNAs were identified in *T. reesei* with high-throughput Solexa sequencing. The differential expression profile of milRNAs under inductive and non-inductive conditions suggests that milRNAs might play a role in *T. reesei* growth and cellulase production. This study will serve as a basis for further functional research of fungal milRNAs and their industrial application.

## Supporting Information

Figure S1 ***T. reesei* cellulase filter paper activity assay.**
(DOCX)

Figure S2 **Length distribution of sRNAs in *T. reesei* IN and CON samples.**
(TIF)

Figure S3 **Annotation of sRNAs of *T. reesei* IN (A) and CON (B).** Pie chart showed the unique or total sequences matched to all categories of rRNA, tRNA, snRNA and snoRNA. The number in bracket showed the reads of the sequences.
(TIF)

Figure S4 **GO classification of potential targets of *T. reesei* milRNAs.** The results were summarized in three main categories as follows: cellular component, molecular function and biological process. In total, 196 genes have been assigned GO terms. In some cases, one gene has multiple terms.
(TIF)

Table S1 **Primers used in S-Poly(T) RT-qPCR assay.**
(XLS)

Table S2 **Measurement of *T. reesei* total RNA.**
(XLS)

Table S3 **Components involved in the sRNA biogenesis of *T. reesei* and two other filamentous fungi.**
(XLS)

Table S4 **Predicted targets of *T. reesei* milRNAs.**
(XLS)
