# Supplementary material for: Identification of microRNA-Like RNAs in the Filamentous Fungus Trichoderma reesei by Solexa Sequencing
Source: PLoS One. 2013 Oct 2;8(10):e76288. doi: 10.1371/journal.pone.0076288 (PMC3788729; doi:10.1371/journal.pone.0076288)
Supplement: Figure S1 — T. reesei cellulase filter paper activity assay. (DOCX) [file pone.0076288.s001.docx]

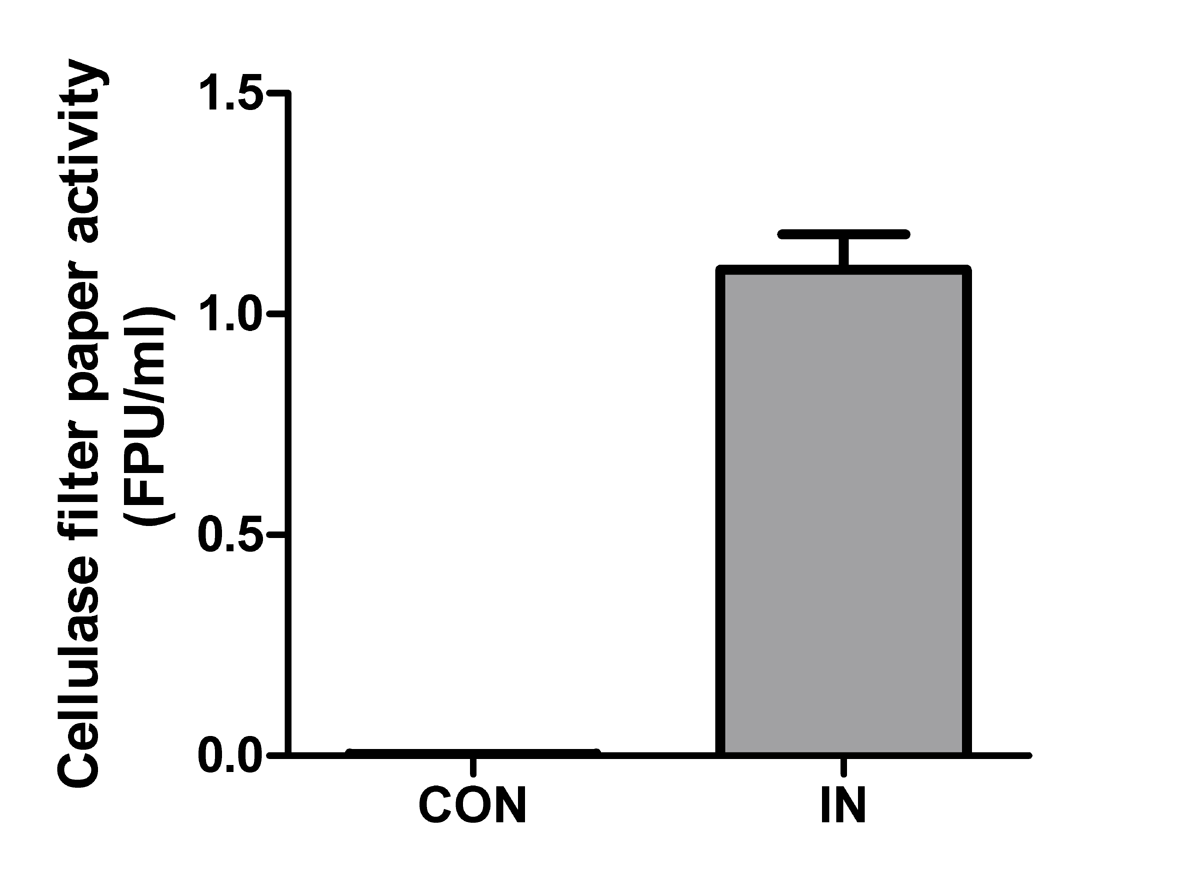


**Additional file 2: Figure S1. The *T. reesei* cellulase filter paper activity assay.** The celulase filter paper activities of the culture supernatant of *T. reesei* CON and IN samples were measured with a method provided by Ghose [[18](#_ENREF_26)]. Analyses were performed in biological triplicate. Each bar represents mean ± SD.

18. Ghose T: **Measurement of cellulase activities**. *Pure Appl Chem* 1987, **59**:257-268.
