# Supplementary figures and images for: Identification of microRNA-Like RNAs in the Filamentous Fungus Trichoderma reesei by Solexa Sequencing
Source: PLoS One. 2013 Oct 2;8(10):e76288. doi: 10.1371/journal.pone.0076288 (PMC3788729; doi:10.1371/journal.pone.0076288)

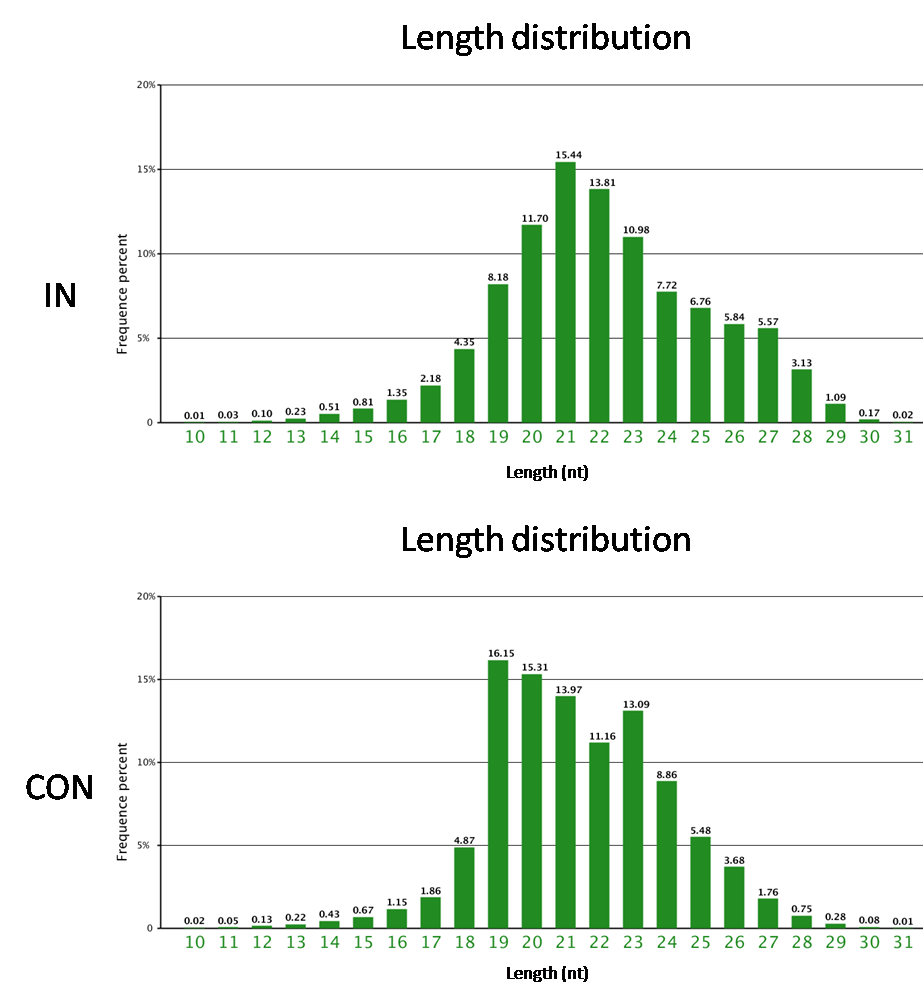

Supplement: Figure S2 — Length distribution of sRNAs in T. reesei IN and CON samples. (TIF) [file pone.0076288.s002.tif]

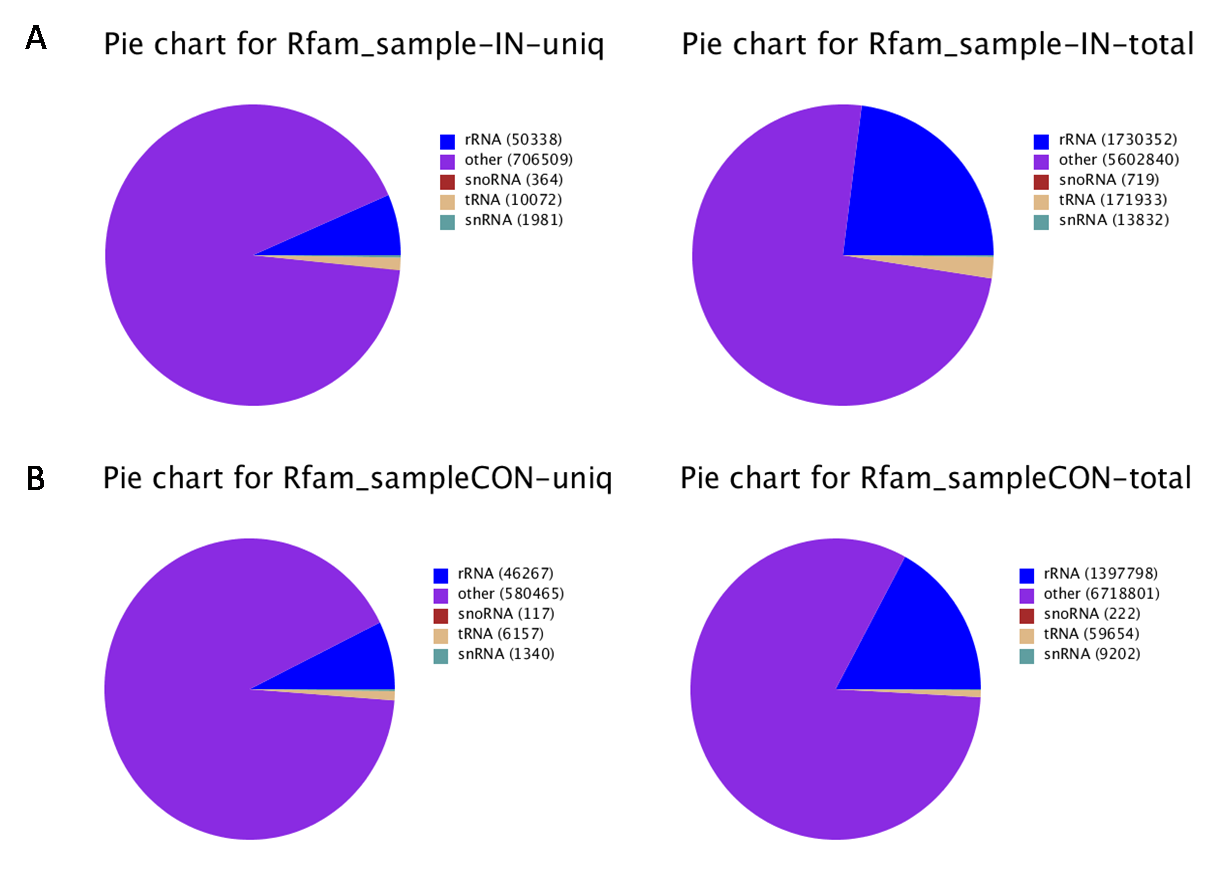

Supplement: Figure S3 — Annotation of sRNAs of T. reesei IN (A) and CON (B). Pie chart showed the unique or total sequences matched to all categories of rRNA, tRNA, snRNA and snoRNA. The number in bracket showed the reads of the sequences. (TIF) [file pone.0076288.s003.tif]

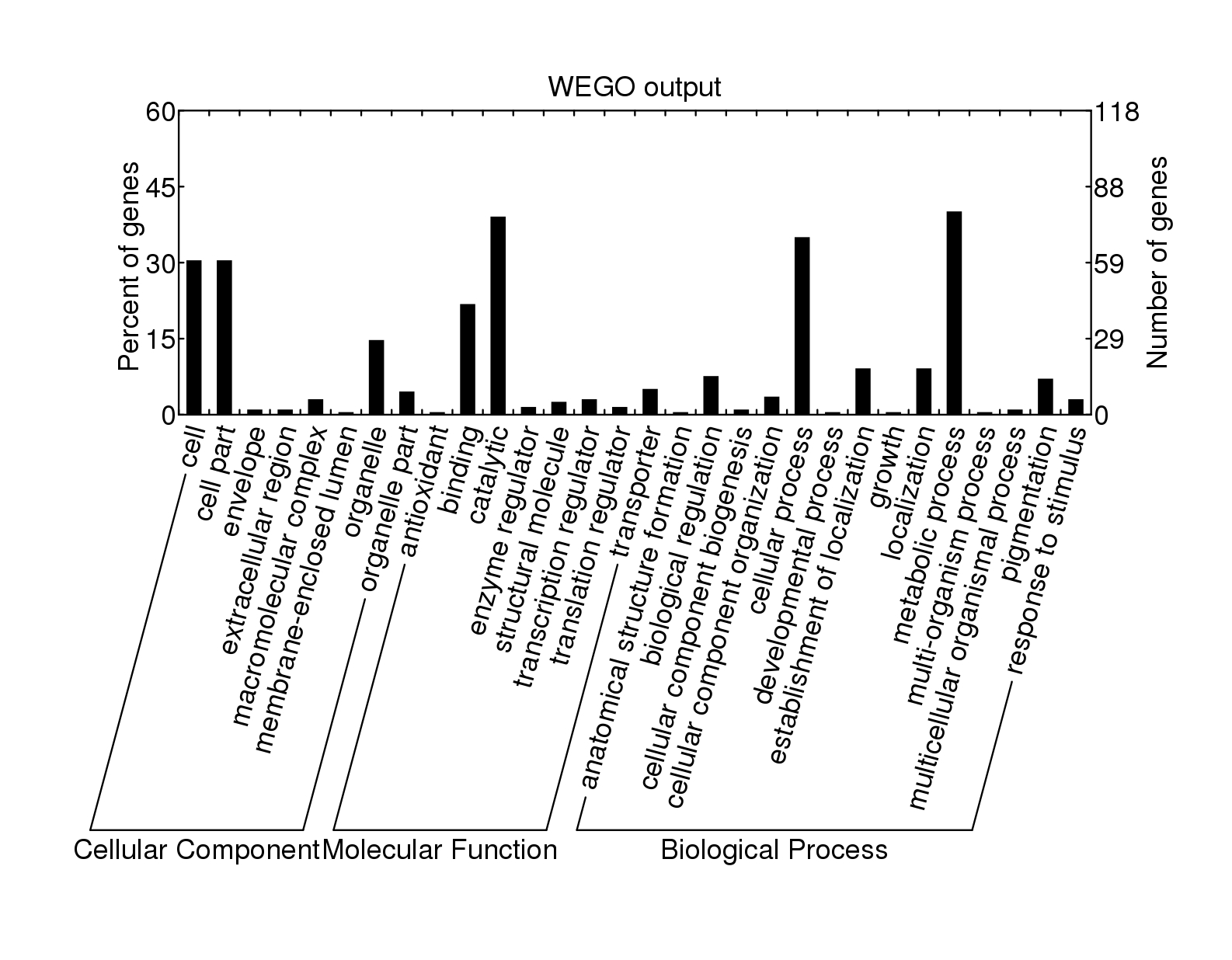

Supplement: Figure S4 — GO classification of potential targets of T. reesei milRNAs. The results were summarized in three main categories as follows: cellular component, molecular function and biological process. In total, 196 genes have been assigned GO terms. In some cases, one gene has multiple terms. (TIF) [file pone.0076288.s004.tif]
